# Factors Influencing the Accessibility of Maternal Health Service in Cambodia

**DOI:** 10.3390/ijerph16162909

**Published:** 2019-08-14

**Authors:** Won Ju Hwang, Yeon Mi Park

**Affiliations:** College of Nursing Science, Kyung Hee University, 26 Kyungheedae-ro, Dongdaemun-gu, Seoul 02447, Korea; hwangwj@khu.ac.kr

**Keywords:** maternal mortality, maternal health services, accessibility

## Abstract

Despite worldwide efforts, maternal and child mortality remains a major health problem in many developing countries. Cambodia’s maternal mortality rate has decreased over recent years through government efforts and support from various international development cooperation agencies. The purpose of this study was to investigate the factors that affected the accessibility of Cambodia’s maternal healthcare services. Data from maternal health service surveys conducted in Battambang, Cambodia in 2012 and 2015 were compared and analyzed. Multiple regression analysis was conducted to identify factors related to the accessibility of integrated maternal healthcare service. The travel time to health centers was found to be related to distance from the health center (ß = 0.031, *p* < 0.001), travel time during the rainy season (ß = 0.166, *p* < 0.001), and travel cost (ß = 0.001, *p* < 0.001), with an explanatory power of 27% (R^2^ = 0.274). Based on these findings, future research and policy should focus on improving accessibility to effective maternal and child healthcare services, to reduce maternal and child mortality. This study is intended to contribute to developing a multi-directional and integrated strategy for access to maternal health services in developing countries.

## 1. Introduction

Although there has been much improvement in maternal and child mortality rates since this issue was adopted by the United Nations (UN) of Millennium Development Goals (MDG) in 2000, this matter remains an important and pressing issue that needs to be addressed in developing countries such as Cambodia. Having actively worked on finding ways to decrease maternal and child mortality rates for 15 years, the UN also included this goal in its Sustainable Development Goals (SDGs) in 2015 as part of the objective of guaranteeing the health and well-being of all people [1]. Indices of mothers’ health conditions are very important for the achievement of these goals because they indicate not only the effectiveness of each country’s healthcare system but also the state of a country’s overall social and financial environment [2].

According to statistics from the 2015 Cambodia Demographic and Health Survey, Cambodia’s maternal mortality rate in 2005 was 472 per 100,000 infants, and the infant death rate was 66 per 1000 infants, showing the grave situation in Cambodia at that time. These rates greatly improved after Cambodia’s inclusion as one of 10 fast-track countries in the Partnership for Maternal, Newborn, and Child Health (PMNCH) program [3]. In 2009, the Cambodian government established the Fast Track Initiative Road Map (FTIRM) for reducing maternal and newborn mortality to accelerate the decrease in death rates and achieve the relevant MDGs; the final goal in this road map was to decrease maternal death rates to 250 per 100,000 infants by 2015. To emphasize the significance of this road map, the government modified it to fit the newly adopted SDGs and initiated the FTIRM 2016–2020. As the road map is linked with the Health Strategic Plan 2016–2020, it will affect the distribution of Cambodia’s yearly budget [4].

The WHO, the World Bank, the United Nations Population Fund, Asian Development Bank, and the United Nations Children’s Fund are the main development cooperation agencies supporting Cambodia, with the WHO connecting and mediating the support from other international health organizations. Furthermore, the United States Agency for International Development has been helping Cambodia develop policies on maternal health, securing the overall system, providing technological and monetary aid, and increasing the accessibility to health services [5]. The goal of all these organizations is to help Cambodia build an infrastructure that improves accessibility to crucial health services, which includes erecting more health facilities, repairing hospitals, instituting family plans, building new maternal facilities, training the essential work force (such as midwives), and educating people regarding the necessity of maternal health care.

As the range of available foreign aid grows ever larger, the significance of research regarding factors that demographically, socioeconomically, or environmentally affect maternal healthcare levels rises in tandem. This is because the healthcare field is affected by developments in all areas of society [6]. Many studies have shown that the maternal death rate is closely related with the mother’s level of education [7,8], hygiene facilities [9], financial standards, the size of the related workforce [10], and the physical distance between mothers and healthcare facilities [11]. Research has also shown that, especially in developing countries, giving birth in professional facilities with the help of skilled birth attendants significantly decreases the maternal mortality rate. The physical distance between mothers’ residences and the closest medical facility is also an important factor, because receiving special treatment immediately after childbirth prevents complications and consequently improves maternal mortality rates [12,13,14,15].

In 2012, with reference to the Cambodian FTIRM, the Korea Foundation for International Healthcare (KOFIH) undertook detailed research on Battambang’s maternal healthcare based on a gap analysis of the original maternal healthcare data. From this, an integrated maternal health project was developed and operated from 2012 to 2016 [16]. Here, integrated means that the program includes various strategies for promoting maternal and child health and community-based activities and strengthening maternal healthcare providers, effective referral systems, and leadership/supervisory systems. The present study aimed to examine the factors that affect maternal healthcare by comparing the 2012 baseline data with the 2015 post-intervention data from Battambang. While this three-year period is considerably short in regard to observing significant differences, the outcome of this research will still likely be helpful in establishing strategies for future maternal and child health programs.

## 2. Methods

### 2.1. Data Collection

This study utilized two different sets of data to compare the status of healthcare in Battambang before and after the implementation of the integrated maternal healthcare project. Specifically, this study used baseline data from the Health Management Department of Seoul National University, which was collected in 2012, to plan for the maternal and child healthcare changes. Also, this study used data that was obtained three years after the WHO and KOFIH intervention took place (in 2015). The first set of data was collected from three Operational Districts (OD) in Battambang Province: Battambang OD, Sangke OD, and Maung Russei OD. The second set of data was collected from sites where the maternal healthcare services took place. To examine the distance between each OD and the rural hospitals involved, three similar towns were chosen as samples. All women who were pregnant at the time and women who gave birth in 2011 from each town were chosen as interviewees. A total of 604 women was interviewed. As the demand for the service is affected by the supply, a study about the current healthcare service and the management system was then conducted. The second set of data from 2015 was derived from questionnaires undertaken in four ODs (Battambang, Maung Russei, Thmarkol, and Sangke), involving a total of 390 families investigated [17]. In Thmarkol OD, the maternal health program was expanded in 2015 (Table 1). Data collection and utilization was approved by K University’s Institutional Review Board (Approval number: KHSIRB-19-063).

### 2.2. Study Questionnaire

Both datasets focused on the following indices: (1) antenatal care (ANC) service, (2) birth delivery and newborn feeding, (3) postpartum care, (4) child vaccination, (5) child health, (6) service acceptability, (7) knowledge, attitude, behavior and practices (KABP) for birth delivery and newborn feeding, (8) KABP for ANC, and (9) challenges for using health services at public health facilities.

The researchers conducted standardized one-on-one interviews with the participants. The surveys were conducted by either research assistants or trained village health volunteers (VHVs), and all interviews were agreed to by the interviewees beforehand. The expected outcomes and the goal of the research were explained in every interview, along with the data collection method and how all research data would be kept confidential. The questionnaire consisted of the following topics: ANC, implementation (giving birth, feeding the newborn, post-birth care, vaccinations, and child’s illnesses), satisfaction with maternal healthcare services at health care centers, knowledge and attitudes (taking care before birth, giving birth and caring for the newborn, nutrition, and vaccinations) of the barriers involved in traveling to health centers and hospitals, receipt of information about maternal and child healthcare, and communication. The questionnaires consist of closed items. In the 2012 data, there are a total of 133 items, including social support from neighbors and individual- and household-level characteristics. In the 2015 data, the questionnaire was reduced to 90 items. All the women from the 2012 survey responded, but the 2015 data does not include any demographic characteristics.

### 2.3. Data Analysis

Epidata 3.1 was used to enter all the survey data. The collected data were analyzed using SPSS 22.0 (SPSS Inc., IBM Company, Seoul, Korea). The sets of data were recoded and then compared due to differences in their original coding, although most parts of the questionnaires were alike. Despite considerable efforts taken during the data collection process, some data could not be used in the coding and analysis process due to poor quality, such as numerous unanswered questions and mis-selected options. Frequencies, percentages, averages, and standard deviations were obtained for the following topics: ANC, implementation of maternal and child healthcare, mothers’ satisfaction with services, and knowledge/attitudes. The significance of the independent variables was examined using either a *t*-test or a chi-squared test. Service accessibility-related factors were analyzed using correlation and regression analysis (confidence interval 95%, *p* < 0.05).

## 3. Results

### 3.1. Characteristics of Integrated Maternal Healthcare Users

In the 2012 baseline data, a total of 604 women in 15 towns in the three ODs were interviewed, with 245 (41%) pregnant at the time and 359 (59%) having an infant less than one year old. In the 2015 study, 390 families in 26 towns in the four ODs were interviewed, comprising a total of 1916 individuals, including 915 non-pregnant women, 921 children, and 27 pregnant women.

### 3.2. Antenatal Care

Regularly scheduled ANC begins in early pregnancy and continues until post-delivery. Antenatal care includes the management of medical, nutritional, and health education. It is intended to reduce maternal and fetal mortality and morbidity, to reduce pre-term births and congenital anomalies, and to promote health monitoring and healthy fetal growth and development. In 2012 and 2015, women who had received ANC once during pregnancy comprised 6 (1.7%) and 5 (1.3%) of the respondents, respectively, showing little difference. However, those who had received care four times comprised 223 (64.3%) and 353 (91.7%) of the respondents, respectively, showing a large increase over the time (Table 2). Among the respondents who did not receive ANC in 2012, there were seven women who had previously given birth and 245 women who were currently pregnant. However, four respondents did not use ANC; one respondent preferred traditional services, the other three respondents did not have enough resources (money or transportation) for these services, and one respondent marked “others” for an answer. In 2015, one respondent answered that she was too busy to use ANC.

### 3.3. Knowledge Levels of Dangerous Symptoms and Service Satisfaction

Knowledge levels of dangerous symptoms were measured in relation to the mother’s knowledge of dangerous symptoms concerning herself after childbirth and in relation to her knowledge of dangerous symptoms concerning her newborn child. An awareness of dangerous symptoms (for both mother and child) is associated with a reduction in complications [18]. This can be inferred from the outcomes of the health program. Through the program, midwives or doctors consult with mothers during prenatal care, are trained in outreach, and deliver basic knowledge of childbirth to mothers.

The knowledge levels concerning dangerous symptoms that mothers may experience after childbirth were shown to be 0.86 ± 0.83 in 2012 and 1.07 ± 0.65 in 2015, and they were not statistically significantly different over time. This is consistent with the results from a provincial health department report, which stated that the technical portion of midwives’ maternal counseling is insufficient and that a manual is needed to improve the professional counseling [17]. On the other hand, the knowledge levels concerning dangerous symptoms that infants may experience were shown to be 2.43 ± 2.09 in 2012 and 2.54 ± 1.36 in 2015 (*p* < 0.001), showing a very slight increase over time.

Satisfaction was measured using a single question about mothers’ satisfaction with the public maternal health services. Satisfaction with the integrated maternal healthcare service increased from 2.90 ± 1.66 in 2012 to 3.21 ± 0.62 in 2015 (Table 3).

### 3.4. Factors Related to the Accessibility of Healthcare Service

#### 3.4.1. Changes in Accessibility

In the 2012 baseline study, the health facilities were located an average of 4.65 km away from respondents, and it took the respondents 28 min (on average) to arrive at the facilities (or 49.5 km during rainy seasons). These travel times are based on the researchers’ calculations. In 2015, these numbers significantly decreased, with facilities located 3.83 km away and the travel time being 17 min on average (37.8 during rainy seasons). Furthermore, travel expenses decreased from 5518 riels to 3593 riels on average. In 2012, people who walked to the facilities comprised 16.9% of the respondents (24.8% during rainy seasons), a figure that decreased to 5.4% (12.1% during rainy seasons) in 2015. Relatedly, the number of people who rode motorcycles or tuk-tuks increased between 2012 and 2015. The respondents’ answers in regard to whether the transportation costs are a burden were 11% and 9% in 2012 and 2015, respectively; however, the respondents’ answers that there is no such burden increased from 36.2% to 73.5% in 2012 and 2015, respectively. Also, the answers for there being no difficulty in finding transportation methods increased from 27.1% in 2012 to 55.5% in 2015 (*p* < 0.001; Table 4).

#### 3.4.2. Relationship between Individual Variables and Distance to Health Centers

A direct proportional relationship was found between the distance to the closest health center and travel time (r = 0.331, *p* < 0.001), travel time during rainy seasons (r = 0.252, *p* < 0.001), transportation expenses (r = 0.461, *p* < 0.001), and trouble finding transportation methods (r = 0.163, *p* < 0.001) (Table 5).

#### 3.4.3. Factors Related to Accessibility of Healthcare Service

Since birth is an imminent event, the time it takes to get to a health center can be an important factor in accessibility, and this time can be influenced by other factors. Factors related to accessibility of healthcare service among the research participants were examined using multiple regression analysis. The multiple regression analysis identified each factor’s independent effects. This study controlled for travel cost burden and difficulty in finding transportation methods. However, we were unable to adjust for demographic factors in this analysis. Time spent getting to health centers was related to distance to the health center (ß = 0.031, *p* < 0.001), travel time during rainy seasons (ß = 0.166, *p* < 0.001), and travel expenses (ß = 0.000, *p* < 0.001). These factors had approximately 27% (R^2^ = 0.274) explanatory power. Furthermore, the problem of multicollinearity did not appear when distance to the health centers, travel time during rainy seasons, travel expenses, and difficulty in finding transportation methods were selected as individual variables (tolerance > 0.1, variance inflation factor < 10) (Table 6).

## 4. Discussion

Countries worldwide are placing ever greater efforts on reaching the UN’s SDGs and MDGs. Particularly in developing countries such as Cambodia, much progress has been made in the past 10 years, but progress in the maternal healthcare field has been more limited in Cambodia, so much so that the government adopted a unique policy regarding this field, the FTIRM 2016–2020. This study aimed to define the factors related to accessibility to healthcare services through comparing data before and after the implementation of an integrated maternal healthcare service by the WHO and KOFIH.

First, although it is not considered the best indicator of positive pregnancy outcomes, receiving ANC lowers mothers’ morbidity rates and death rates [19,20]. This study’s results showed that the rate of receiving ANC rose due to the integrated maternal healthcare service, similar to the results from projects in Volta, Ghana, and in Kibaha, Tanzania [21,22]. In fact, the rate for receiving ANC was found to be higher than the Cambodian national ANC average [3]. Mother’s parity [23,24,25], mother’s education level [26,27,28,29], household income [3,26,29,30,31,32], differences between cities and rural areas or local communities [3,18,23,33], and father’s education level [28,34] have been reported as other significant factors affecting the quality of maternal healthcare services during pregnancy. Other factors affecting service usage that have been identified include physical distance and a scarcity of healthcare providers and health infrastructures for women living in rural areas [35,36]. Thus, future research and further analysis are required to confirm whether the ANC rate increase found in this study was really due to the integrated maternal healthcare service.

Second, service satisfaction and education levels regarding infant care increased significantly. One of the major goals of maternal healthcare projects in developing countries is to improve infant healthcare, which is mostly performed alongside ANC and is used as an index reflecting healthcare service provision and user satisfaction. Continuous ANC along with nutrition advice, breast-feeding, vaccine provision, healthcare, and after-pregnancy education lead to higher levels of knowledge and service satisfaction. The KOFIH has been working on training more professional midwives and improving the education environment through continuous financial support since the completion of a midwife training center in June 2013. Furthermore, this organization has been educating and monitoring VHVs, who educate locals on basic healthcare. Strategic efforts such as these appeared to have increased the education levels of locals and their service satisfaction. Also, the results of this study showed meaningful growth in knowledge levels concerning dangerous infant-related health symptoms, which was one important factor reported to affect infant mortality in a 2016 comparison study conducted in Indonesia [18]. Although this increased knowledge does not necessarily indicate higher overall education levels, identifying symptoms in the early stages of pregnancy and increased knowledge regarding infant care contribute to preventing infant death.

Third, lack of access to local healthcare services, including antenatal care, has been consistently reported as one of the major contributors to maternal mortality and morbidity [19,37]. Particularly in developing countries, where transportation infrastructure is often underdeveloped, issues in relation to physical barriers such as location, distance involved in traveling, travel time, and transportation to health facilities has long been investigated by researchers [28,32,34,38,39,40,41]. For these reasons, much non-governmental organization activity is focused on creating a healthcare infrastructure based on service accessibility. The KOFIH has also reinforced their referral system through financially supporting patients, providing maternal child wards in hospitals, and providing tuk-tuks as a method of transportation. As a result, the average distance to healthcare facilities, travel time, and cost have decreased. The results of this study also showed that travel time during the dry season was more than twice as short as that during the rainy season. This means that unlike dry weather conditions in the rainy season, special intervention is needed to improve accessibility.

Cost is another major factor that must be considered in terms of healthcare service accessibility, as it may lead people to finding alternatives to healthcare facilities. When an individual cannot afford treatment or an ambulance, accessibility drops, leading to an increase in dependence on untrained local traditional birth attendants [42]. This may lead to complications and infections in the mother and child [43].

Overall, the results of this study clearly showed that distance to healthcare centers, travel time, travel cost, and transportation methods were important factors affecting healthcare service accessibility. Therefore, in developing countries such as Cambodia, it is important to address these issues and the related financial burdens by carefully planning facility locations in a way that considers individuals’ actual travel time to ensure the provision of effective healthcare for mothers and their children.

## 5. Conclusions

These findings strongly suggest that distance to healthcare centers, travel cost, travel time, and transportation methods are all important factors affecting accessibility. However, maternal healthcare is problematic beyond the realm of just healthcare. It is important to simultaneously consider other factors such as alleviating poverty, developing the infrastructure (including facilities and the workforce) to increase accessibility to healthcare services, and increasing women’s education levels, social status, delivery culture, and awareness of dangerous symptoms. Therefore, when developing plans for maternal healthcare, a serious in-depth investigation of the relevant factors is necessary, including quantitative research on each factor’s inter-related effects on maternal healthcare provision. In addition, the resulting strategy developed for integrated maternal healthcare must be customized to the needs of specific societies and communities.

However, this study has several limitations. First, poor data quality in some cases meant that a number of data could not be used in the coding and analysis processes. Second, as this study involved a comparison of data with only a three-year gap between the datasets, the results are not necessarily related or conclusive. Finally, the possibility of response bias cannot be excluded because the interviewers’ attitudes and manners could have affected the responses. It is recommended that future studies take these factors into account.

## Figures and Tables

**Table 1:** Data for the Operational Districts by year.

Year	Total	Battambang	Maung Russei	Sangke	Thmarkol *
	*N* (%)	*N* (%)	*N* (%)	*N* (%)	*N* (%)
2012	604 (100)	64 (10.6)	247 (40.9)	293 (48.5)	-
2015	390 (100)	105 (27)	131 (33.6)	76 (19.4)	78 (20)

* In Thmarkol OD, the maternal health program was expanded in 2015.

**Table 2:** Distribution of antenatal care service in 2012 and 2015 (*N* = 576).

Variables	2012 Number (%)	2015 Number (%)	X^2^	*p*
ANC (1 time)	6 (1.7)	5 (1.3)	95.92	<0.001
ANC (2 times)	5 (1.4)	6 (1.6)		
ANC (3 times)	118 (33.5)	21 (5.5)		
ANC (4 times)	223 (63.4)	353 (91.7)		

ANC = antenatal care.

**Table 3:** Distribution of mothers’ knowledge of dangerous symptoms and satisfaction with maternal services at healthcare centers in 2012 and 2015 (*N* = 576).

Variables		2012	2015	t	*p*
Knowledge	concerning dangerous symptoms after childbirth in the mother	0.86 ± 0.83	1.07 ± 0.65	−0.98	0.324
	concerning dangerous symptoms in infants	2.43 ± 2.09	2.54 ± 1.36	−4.48	<0.001
Satisfaction with maternal healthcare services at healthcare centers		2.90 ± 1.66	3.21 ± 0.62	−2.30	0.022

**Table 4:** Distribution of factors related to accessibility of maternal and child health services in 2012 and 2015 (*N* = 994).

Variables	2012	2015	t or χ^2^	*p*
Distance to health center (km)	4.65 ± 5.24	3.83 ± 5.13	2.42	0.015
Travel time (min)	28.2 ± 27.6	17.4 ± 33.0	5.50	<0.001
Travel time during rainy season (min)	49.5 ± 66.2	37.8 ± 117.0	1.78	0.075
Travel cost (riel)	5518.16 ± 5767.8	3592.0 ± 7038.8	4.463	<0.001
Transportation methods (%)				
Walking	16.9	5.4	38.18	<0.001
Car	0.7	0.3		
Motorcycle/tuk-tuk	66.4	80.0		
Horse cart	0.0	0.5		
Bicycle	14.9	13.6		
Boat	0.8	0.3		
Other	0.3	0.0		
Transportation methods during rainy season (%)				
Walking	24.8	12.1	154.97	<0.001
Car	2.6	0.5		
Motorcycle/tuk-tuk	40.7	67.2		
Horse cart	0.2	5.4		
Bicycle	9.3	11.3		
Boat	18.2	2.8		
Other	4.1	0.8		
Travel cost is a burden (%)				
Yes	11.0	9.0	138.83	<0.001
A little	52.8	17.5		
No	36.2	73.5		
Difficulty in finding transportation methods (%)				
Yes	17.6	16.2	87.86	<0.001
A little	55.3	28.3		
No	27.1	55.5		

**Table 5:** Correlations between distance to health centers and independent variables (*N* = 994).

Variables	1	2	3	4	5	6
1. Distance to health centers	-					
2. Travel time in minutes	0.331 *	-				
3. Travel time during rainy season	0.252 *	0.360 *	-			
4. Travel cost	0.461 *	0.325 *	0.198 *	-		
5. Travel cost is a burden	0.217 *	0.172 *	0.152 *	0.202 *	-	
6. Difficulty in finding transportation methods	0.163 *	0.128 *	0.107 *	0.163 *	0.517 *	-

* *p* < 0.001, two-tailed.

**Table 6:** Multiple regression analysis of the effects of selected variables on travel time in minutes (*N* = 994).

Variables	β	t	*p*
Distance to health centers	0.013	4.011	<0.001
Travel time during rainy seasons	0.166	12.272	<0.001
Travel cost	0.000	4.641	<0.001
Travel cost is a burden	0.053	1.531	0.126
Difficulty in finding transportation methods	0.020	0.560	0.576
Intercept	0.280		
F	70.169		
R^2^	0.278		
Adjusted R^2^	0.274		
*p*	<0.001		

## References

[1] United Nations Transforming Our World: The 2030 Agenda for Sustainable Development. https://sustainabledevelopment.un.org/post2015/transformingourworld.

[2] OECD/World Health Organization (2012). Health at a Glance: Asia/Pacific 2012.

[3] National Institute of Statistics, Directorate General for Health, and ICF International, 2015. Cambodia Demographic and Health Survey 2014. Phnom Penh, Cambodia and Rockville, Maryland, USA: National Institute of Statistics, Directorate General for Health, and ICF International. https://dhsprogram.com/pubs/pdf/fr312/fr312.pdf.

[4] Kingdom of Cambodia Ministry of Health Fast Track Initiative Road Map for Reducing Maternal and Newborn Mortality 2016–2020. http://cambodia.unfpa.org/sites/default/files/pub-pdf/FTIRM_2016_2020_enlgish.pdf.

[5] USAID Global Health. https://www.usaid.gov/cambodia/global-health.

[6] Hall J.J., Taylor R. (2003). Health for all beyond 2000: The demise of the Alma-Ata Declaration and primary health care in developing countries. Med. J. Aust..

[7] Markovitz B.P., Cook R., Flick L.H., Leet T.L. (2005). Socioeconomic factors and adolescent pregnancy outcomes: Distinctions between neonatal and post-neonatal deaths?. BMC Public Health.

[8] Gakidou E., Cowling K., Lozano R., Murray C.J. (2010). Increased educational attainment and its effect on child mortality in 175 countries between 1970 and 2009: A systematic analysis. Lancet.

[9] Cheng J.J., Schuster-Wallace C.J., Watt S., Newbold B.K., Mente A. (2012). An ecological quantification of the relationships between water, sanitation and infant, child, and maternal mortality. Environ. Health.

[10] Bayati M., Vahedi S., Esmaeilzadeh F., Kavosi Z., Jamali Z., Rajabi A., Alimohamadi Y. (2016). Determinants of maternal mortality in Eastern Mediterranean region: A panel data analysis. Med. J. Islam. Repub. Iran..

[11] Karra M., Fink G., Canning D. (2017). Facility Distance and Child Mortality: A Multi-Country Study of Health Facility Access, Service Utilization, and Child Health Outcomes. Int. J. Epidemiol..

[12] World Health Organization (2005). The World Health Report 2005-Make Every Mother and Child Count.

[13] Johnson K.C., Daviss B.A. (2005). Outcomes of planned home births with certified professional midwives: Large prospective study in North America. BMJ.

[14] Janssen P.A., Lee S.K., Ryan E.M., Etches D.J., Farquharson D.F., Peacock D., Klein M.C. (2002). Outcomes of planned home births versus planned hospital births after regulation of midwifery in British Columbia. Can. Med. Assoc. J..

[15] Gazi R., Goodburn A.E., Chowdhury M., Marshall T., Graham W. (2000). Training traditional birth attendants in clean delivery does not prevent postpartum infection. Health Policy Plan..

[16] Oh J.H., Kang M.A., Kim Y. (2012). Developing a Program on Maternal and Child Health Care in Battambang Province, Cambodia.

[17] Veuong B., Kuch K. (2016). Report of the Survey on Reproductive Health-Youth Maternal Child Health and Nutrition in Battambang 2015.

[18] Abdullah A., Hort K., Butu Y., Simpson L. (2016). Risk factors associated with neonatal deaths: A matched case–control study in Indonesia. Glob. Health Action.

[19] Finlayson K., Downe S. (2013). Why Do Women Not Use Antenatal Services in Low and Middle-Income Countries? A Meta-Synthesis of Qualitative Studies. PLoS Med..

[20] Moore N., Blouin B., Razuri H., Casapia M., Gyorkos T.W. (2017). Determinants of First Trimester Attendance at Antenatal Care Clinics in the Amazon Region of Peru: A Case-Control Study. PLoS ONE.

[21] Korea Foundation for International Healthcare (2016). Evaluation Report of Intermediate Evaluation of Maternal and Child Health Promotion Project in Ghana.

[22] Korea Foundation for International Healthcare (2014). Outcome Report of Consulting Using Maternal and Child Health Center, Tanzania.

[23] Mekonnen Y., Mekonnen A. (2013). Factors Influencing the Use of Maternal Healthcare Services in Ethiopia. J. Health Popul. Nutr..

[24] Celik Y., Hotchkiss D.R. (2000). The socio-economic determinants of maternal health care utilization in Turkey. Soc. Sci. Med..

[25] Bathia J.C., Cleland J. (1995). Determinants of Maternal Care in a Region of South India. Health Transit. Rev..

[26] Addai I. (2000). Determinants of use of maternal-child health services in rural Ghana. J. Biosoc. Sci..

[27] Jejeebhoy S.J. (1996). Women’s Education, Autonomy and Reproductive Behaviour: Experience from Developing Countries.

[28] Sunil T., Rajaram S., Zottarelli L.K. (2006). Do individual and program factors matter in the utilization of maternal care services in rural India? A theoretical approach. Soc. Sci. Med..

[29] Celik Y. (2000). The socio-economic determinants of alternative sources of antenatal care in Turkey. Int. J. Health Plan. Manag..

[30] Bazant E.S., Koenig M.A., Fotso J.C., Mills S. (2009). Women’s Use of Private and Government Health Facilities for Childbirth in Nairobi’s Informal Settlements. Stud. Fam. Plann..

[31] Fan L., Habibov N.N. (2009). Determinants of maternity health care utilization in Tajikistan: Learning from a national living standards survey. Health Place.

[32] Gage A.J., Calixte M.G. (2006). Effects of the physical accessibility of maternal health services on their use in rural Haiti. Popul. Stud..

[33] Magadi M., Diamond I., Rodrigues R.N. (2000). The determinants of delivery care in Kenya. Biodemography Soc. Biol..

[34] Pebley A.R., Goldman N., Rodríguez G. (1996). Prenatal and Delivery Care and Childhood Immunization in Guatemala: Do Family and Community Matter?. Demography.

[35] Kyomuhendo G.B. (2003). Low Use of Rural Maternity Services in Uganda: Impact of Women’s Status, Traditional Beliefs and Limited Resources. Reprod. Health Matters.

[36] Wall L.L. (1996). Obstetric fistulas in Africa and the developing world: New efforts to solve an age-old problem. Women’s Health Issues.

[37] Campbell O.M., Graham W.J. (2006). Lancet Maternal Survival Series Steering Group. Strategies for Reducing Maternal Mortality: Getting on with What Works. Lancet.

[38] Sagna M.L., Sunil T. (2012). Effects of individual and neighborhood factors on maternal care in Cambodia. Health Place.

[39] Amooti-Kaguna B., Nuwaha F. (2000). Factors influencing choice of delivery sites in Rakai district of Uganda. Soc. Sci. Med..

[40] Houweling T.A., Ronsmans C., Campbell O.M., Kunst E.A. (2007). Huge poor–rich inequalities in maternity care: An international comparative study of maternity and child care in developing countries. Bull. World Health Organ..

[41] Rockers P.C., Wilson M.L., Mbaruku G., Kruk M.E. (2009). Source of Antenatal Care Influences Facility Delivery in Rural Tanzania: A Population-Based Study. Matern. Child Health J..

[42] Bigdeli M., Annear P.L. (2009). Barriers to access and the purchasing function of health equity funds: Lessons from Cambodia. Bull. World Health Organ..

[43] Yanagisawa S., Oum S., Wakai S. (2006). Determinants of skilled birth attendance in rural Cambodia. Trop. Med. Int. Health.

